# Computer versus paper insulin protocol for managing hyperglycemia in three ICUs

**DOI:** 10.1186/cc14448

**Published:** 2015-03-16

**Authors:** A Peckham

**Affiliations:** 1 Oregon Health & Science University, Portland, OR, USA

## Introduction

The purpose of this study was to compare a computer protocol against a paper protocol in managing three domains of glucose control. Hyperglycemia is common in critically ill patients, and their risk of death is associated with hyperglycemia, hypoglycemia, and glucose variability. A safe and effective insulin protocol must minimize hyperglycemia and glucose variability while also avoiding hypoglycemia. Computer-based insulin protocols promise better performance by adjusting to each individual's sensitivity to insulin.

## Methods

This is a historical cohort study with 759 patients admitted to three ICUs (medical/cardiac, trauma, and neuroscience) at an academic tertiary care hospital. All adult patients from January 2012 to October 2013 on one of two continuous insulin protocols for at least 8 hours were included. At the start of the study period the paper protocol in use (Adult ICU) had a target glucose of 140 to 180 mg/dl and was used for any patient with a glucose higher than 180 mg/dl. In June 2013 this was replaced by a computer-based insulin protocol (EndoTool) that had the same criteria for initiation and had a target glucose of 150 mg/dl. The primary exposure was the insulin protocol, and the primary outcome was performance in maintaining glucose control.

## Results

The median glucose in the EndoTool group (141.5 mg/dl) was lower than in the Adult ICU group (159.9 mg/dl) (*P *< 0.0001). The standard deviation of glucose in the EndoTool group (32.3 mg/ dl) was lower than the Adult ICU group (39.5 mg/dl) (*P *= 0.0001). The proportion of patients in each group with 10% or higher of measurements at a severe hyperglycemia level (≥200 mg/dl) in the EndoTool group (35.2%) was lower than the Adult ICU group (64.1%) (*P *< 0.0001). The proportion of patients who had at least one moderate hypoglycemic measurement (<70 mg/dl) was not significantly different between the EndoTool group versus the Adult ICU group (11.73% vs. 9.3%, respectively; *P *= 0.34). However, there was a higher overall incidence of hypoglycemia in the EndoTool group (5.65 hypoglycemic measurements/100 person-protocol days) compared with the Adult ICU group (3.43/100 person-protocol days) (RR = 1.65, 95% CI = 1.09 to 2.45, *P *= 0.014). Severe hypoglycemia (<40 mg/dl) was rare, only occurring in 1/179 (0.56%) in the EndoTool group and 4/580 (0.69%) in the Adult ICU group.

## Conclusion

Patients on the computer protocol had a lower median glucose, less variability, and less hyperglycemia than patients on the paper protocol. There was a higher risk of moderate but not severe hypoglycemia in the computer group.

